# Assembly Modulated by Particle Position and Shape: A New Concept in Self-Assembly

**DOI:** 10.3390/ma10111291

**Published:** 2017-11-10

**Authors:** Joe W. Tavacoli, Julien Heuvingh, Olivia Du Roure

**Affiliations:** 1 Physique et Mécanique des Milieux Hétérogènes, CNRS, Université Pierre et Marie Curie, Université Paris Diderot, ESPCI Paris, PSL Research University, 75005 Paris, France; julien.heuvingh@espci.fr (J.H.); olivia.duroure@espci.fr (O.D.R.); 2 Department of Energy of Conversion and Storage, Technical University of Denmark, 4000 Roskilde, Denmark

**Keywords:** self-assembly, magnetic systems, MEMS, microrobotics

## Abstract

In this communication we outline how the bespoke arrangements and design of micron-sized superparamagnetic shapes provide levers to modulate their assembly under homogeneous magnetic fields. We label this new approach, ‘assembly modulated by particle position and shape’ (APPS). Specifically, using rectangular lattices of superparamagnetic micron-sized cuboids, we construct distinct microstructures by adjusting lattice pitch and angle of array with respect to a magnetic field. Broadly, we find two modes of assembly: (1) immediate 2D jamming of the cuboids as they rotate to align with the applied field (rotation-induced jamming) and (2) aggregation via translation after their full alignment (dipole-dipole assembly). The boundary between these two assembly pathways is independent on field strength being solely a function of the cuboid’s dimensions, lattice pitch, and array angle with respect to field—a relationship which we capture, along with other features of the assembly process, in a ‘phase diagram’. In doing so, we set out initial design rules to build custom made assemblies. Moreover, these assemblies can be made flexible thanks to the hinged contacts of their particle building blocks. This flexibility, combined with the superparamagnetic nature of the architectures, renders our assembly method particularly appropriate for the construction of complex actuators at a scale hitherto not possible.

## 1. Introduction

The controlled fabrication of nano/micro meter-scale objects is one of today’s central goals in science and technology—one required for the development of nanotechnology and the expansion of microtechnology [1]. Because said objects fall awkwardly between the sizes that can be manipulated by chemistry and those that can be manipulated by standard manufacturing, the most promising strategy for their fabrication is self-assembly, that is, the autonomous organization of components into structures [2].

Because of their potential to be remotely manipulated, extremely flexible building blocks for self-assembly are magnetic nano- and micro-particles. Pioneered by de Gennes and Pincus, the study of their assembly has evolved into a rich scientific landscape [3]. Recent advances include directing such particles into complex 2D shapes via magnetizable media templating, assembling a zoo of uniform architectures from mixture of magnetic and non-magnetic beads and the use of magnetic precession to dynamically ensemble magnetic Janus beads into fluctuating and morphology altering microtubules [4,5,6,7,8]. Moreover, shape anisotropy has been combined with magnetism to make structures with complex actuation responses to even homogenous magnetic fields. For instance, Erb et al. have fabricated shape changing materials by varying the orientation of magnetic platelets in individual layers of a composite structure and just recently patchy magnetic cubes have been assembled into reconfigurable microstructures that have promising future applications in cell transport and release [9,10].

Though the role of particle design in guiding assembly is well documented, the use of particle prepositioning is less so, being reserved for arbitrary, singular patterns of particles of the same shape—or on a few selected ones typically positioned by magnetic or laser tweezers [11,12].

In this communication we propose, and perform proof-of-concept experiments on, a new concept in self-assembly that couples both shape anisotropy and particle preposition to assembly via magnetic interactions. This process, which we call APPS (Assembly modulated by Particle Position and Shape), relies on a soft lithography to generate micron-sized supermagnetic (SP) particles whose shape can be arbitrarily designed and initial position controlled on a 2D lattice. Through the application of a magnetic field, these particles can be assembled into bespoke structures.

Whilst micro patterned molds have been used previously to modulate the self-assembly of particles and to print particles directly into advanced hierarchal structures on the nano- and microscale, the final particle arrangements dictated by the molds are the ones desired and also static. In APPS, the arrangement is the starting position of the particles prior to assembly [13,14,15,16,17].

Accordingly, APPS is well placed to meet a major construction challenge in robotics to build articulated actuators that allows semi-autonomous motion of parts and many motional degrees of freedom at the colloidal/granular scale [18,19,20,21]. Such actuators, or microrobots, will enable minimally invasive diagnosis and treatment inside the human body, biological studies in microfluidic channels, desktop micromanufacturing applications, and the creation of systems to build still more minute and complex architectures [21,22,23]. The main obstacle to the fabrication of these structures lies with difficulties in assembling bespoke units on the order of single microns, precisely—and hence exact, customized assembly has been reserved for building blocks on the hundreds of microns to millimeter scale [21,24,25,26,27]. APPS circumvents this restriction because it allows custom-made micro parts as small as 2 microns to be assembled to sub-micron precision through judicious choice of their starting arrangement. Moreover, the particle dimension limits Brownian motion allowing pre-positioning to be preserved during the assembly. Additionally, microstructures formed from APPS are SP and can therefore be remotely operated in a range of media. Hence, they can be suitable for application as microrheological probes, devices in precise single-particle drug delivery operations, and as microelectromechanical systems (MEMS) [28].

**Figure 1:**
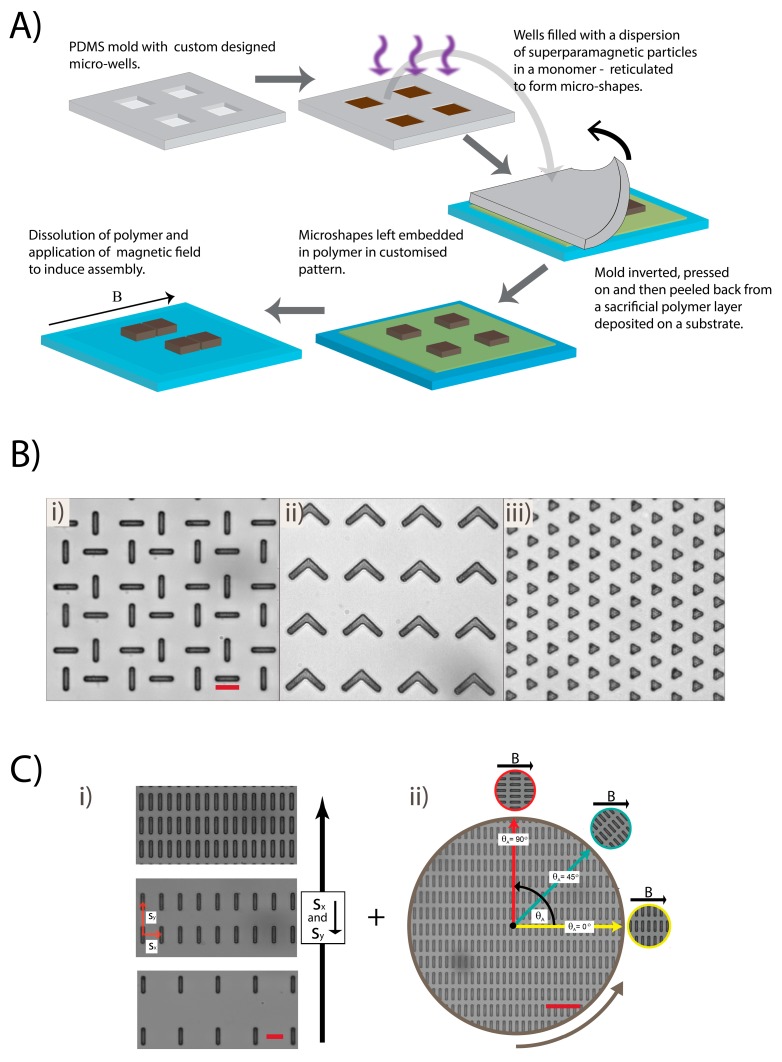
(**A**) The underlying method facilitating APPS: fabrication of SP micron-sized particles and there extraction onto a substrate. (**B**) SP rectangles (i), ‘boomerangs’ (ii), and triangles (iii) developed and extracted by the described method in three different prearrangements. Scale bars are 20 μm. (**C**) The assembly variables studied: array pitch (i) and angle of the array with respect to a magnetic field maintained in the *x* direction (ii). Scale bars are 20 and 60 μm for (i) and (ii), respectively.

We demonstrate the potential of APPS through a set of simple experiments performed on SP micron-sized cuboids. Though simple, these experiments highlight the power of this new approach—one that will inevitably be used to make advanced assemblies fit for application in the future. Before we outline these experiments, it is instructive to detail, briefly, the soft lithography-based fabrication and extraction processes that enable it (Figure 1A) [29,30].

Polydimethylsiloxane (PDMS) molds holding patterns of microwells are fabricated as standard, i.e., templated by a custom designed Cr mask. The microwells are then filled with a dispersion of silica-coated SP colloids in the monomer ethoxylated trimethylolpropane triacrylate (ETPTA), which is reticulated to leave composite SP micron-sized particles with a form and arrangement set by the PDMS mold. The silica-coated SP colloids contain iron oxide nanoparticles whose single, nano-sized magnetic domains remain discrete during the reticulation process. Accordingly, the fabricated micron-sized particle are themselves SP and their magnetization value is a reflection of the wt % of the iron oxide nanoparticles they hold. In a previous study, we deduced a nominal volume of susceptibility of 0.63—linear up to 10 mT, a saturation magnetization of 4 × 10^4^ A·m^−1^ and a magnetization of 1.6 × 10^4^ A·m^−1^ at 40 mT [29,31,32]. The nominal magnetization was reaffirmed by calculating the magnetization of micron-sized cylinders via their Stokes drag under a range of magnetic field gradients [29]. To remove the particles from the mold, it was placed face down on sticky homogenously-thick sacrificial polymer layer (blade-cast on a glass microscope slide) and gently peeled back, thereby extracting the particles in their custom arrangements. To release the particles in their bespoke positions, a PDMS frame 500–800 μm thick is positioned around the pattern, a drop of a viscous 1:1 by volume mixture of ethanol and ethanediol loaded on top and the framework sealed with a glass cover slip. This configuration prohibits evaporation and flow of the solvent while the solvent itself slowly dissolves the sacrificial polymer layer, providing ample time for the application of the magnetic field before particle release. Significantly, the uniform depth and surface of the polymer promotes its homogenous dissolution and the synchronized release of the particles—allowing their prepositioning to guide their assembly. Furthermore, because the particles are tens of microns in size, Brownian fluctuations are negligible and they move and assemble solely because of their magnetic properties.

With this protocol, practically any 2D shape can be made down to sizes of 2 microns, patterned with submicron precision onto a surface of choice (Figure 1B). Laying down SP particles with such exactness, with exquisite control of their shape, provides the basis for APPS. On application of a magnetic field and gentle dissolution of the sacrificial polymer, the shapes assemble directed by both their customized arrangement and form(s) to leave architectures whose design is determined by these factors. We have already demonstrated that SP micron-sized circular cylinders starting from a square lattice arrange into a face-centered square lattice of dimers and here we expand the concept to SP micron-sized cuboids (henceforth referred to only as cuboids) that have a complex response to magnetic fields because they are subject to both magnetic torques and dipolar attractions/repulsions [29]. We focus specifically on cuboids prepositioned on a rectangular array with their long axis oriented in the same direction. We systematically study the impact of array dimensions and orientation of the array with respect to a magnetic field, revealing the mechanisms of self-assembly and resulting structures formed (Figure 1C).

## 2. Results

### 2.1. Self-Assembly Guided by a Magnetic Field Perpendicular to the Long Axis of the Cuboids

We first extract the cuboids (5 μm × 20 μm) in regular rectangular lattices of three different center-to-center separations *s*, along the *x* and *y* axis. Assembly is induced through the application of a 5 mT homogeneous field perpendicular to their long axis (*θ_A_* = 0°), with simultaneous dissolution of the polymer layer they are embedded within. On application of the field, all cuboids are subject to magnetic torque and consequently proceed to rotate their long axis to align with the field and rotating left or right during the alignment process is equally energetically favorable.

**Figure 2:**
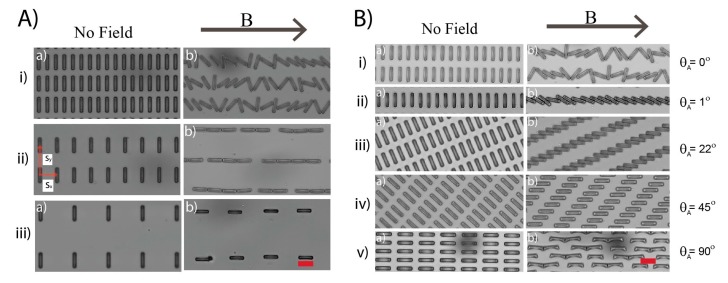
(**A**) Bright-field micrographs of cuboids (5 μm × 20 μm) in starting patterns (a) with array pitches *s_x_* and *s_y_* of 13 and 32 μm (i), 26 and 44 μm (ii), and 32 and 64 μm (iii). On application of a 5 mT homogeneous field (b) the cuboids assemble into disordered linear chains (i), regular linear chains (ii), and remain as singlets (iii), respectively. Corresponding time differences between (a) and (b) for (i–iii) are 6, 59.66 and 128.66 s. (**B**) The structures formed at short times under field at *θ_A_* = 0, 1, 22, 45, and 90° with a time interval between panels (a) and (b) of 7, 8, 10, 1, and 4 s respectively. A 5 mT homogeneous magnetic field was applied at *θ_A_* = 0 and 1°, an 8 mT field for *θ_A_* = 22° and a 20 mT field was utilized at *θ_A_* = 45 and 90°. Scale bars are 20 μm.

Under these conditions, there is a transition between two assembly processes as lattice spacing is increased (Figure 2A). In the most tightly packed lattice, (Figure 2A(i), *s_x_* = 13 μm), the cuboids are unable to fully align with the field before their rotation is blocked by the contact with neighboring particles. This jamming, coupled with their bidirectional rotation, leads to disordered, yet linear, chains of cuboids positioned at various angles. Each of these chains contains all the cuboids in a row due to their simultaneous involvement in this single assembly step, mediated by their rotation. These chains, and other structures formed by this process, remain intact after field release. This is to be expected as crosslinked ETPTA—the polymeric material of the cuboids—contains no charge groups and electrostatic repulsion between particles is therefore absent, allowing permanent connection with van der Waals forces. Despite this, the structures are not necessarily rigid and flexibility at particle-particle contacts can be engineered via particle design. (cf. Section 3.2).

When the lattice spacing is increased so that the center-to-center distance is greater than the longest dimension of the cuboid, there is sufficient separation along the *x*-axis to allow the cuboids to fully align with the field (Figure 2A(ii), *s_x_* = 26 μm). In this condition, the cuboids first rotate fully and then attract each other via magnetic dipolar interactions to form chains comprising of end-to-end contacts leading to segments of two to four cuboids over a minute. These chains fully align with the field and are 1D, as has been previously reported when magnetic forces are vastly greater that Brownian ones [33,34,35]. The process of aggregation is time dependent as the chains mature to give longer segments (cf. Supplementary Material, Figure S1) and consistent with previous studies, growth is faster under higher magnetic fields [36]. This is in contrast to the jamming assembly process where any field strength will be sufficient to induce rotation of the cuboids and the formation of jammed chains spanning the whole sample. When *s_x_* is increased still further (Figure 2A(iii), *s_x_* = 32 μm), the attraction between the cuboids after rotation is no longer sufficient to trigger assembly. The threshold between the formation of end-to-end chains and non-interaction of the cuboids depends, of course, on the strength of the field.

In summary, depending on the dimensions of the cuboid to the array pitch, *s_x_*, two different assembly processes are evident upon a perpendicular application of the field to their long axis: one based on jamming between particles while rotating and one based on attraction between fully aligned particles. We label these two assembly processes ‘rotation-induced jamming’ and ‘dipole-dipole assembly’, respectively.

### 2.2. Varying the Initial Orientation of the Cuboids

Because assembly in APPS is induced by SP interactions, identical starting patterns can proceed to form distinct architectures simply by changing the angle of the cuboids lattice with respect to a homogeneous magnetic field, *θ_A_*, further increasing the flexibility of this approach.

Again, we demonstrate this potential using the cuboids arranged in simple rectangular lattice and rotating the lattice with respect to an applied field. This is the same lattice that formed disordered linear chains (Figure 2A(i)), when the magnetic field is placed perpendicular to the principal axis of the cuboids (i.e., *θ_A_ =* 0°). The first striking transition in the assembly happens when the field direction is slightly misaligned with the principal axis (*θ_A_* = 1°). In this case, instead of experiencing bi-directional rotation, all cuboids turn in the same direction to produce a regular jammed chain with a ‘stair case structure’ (Figure 2B(ii)). This transition is quite straightforward, as in the case of perpendicular field, the cuboids’ initial conformation corresponds to an energy maxima leading to bi-directional rotation, while at *θ_A_* = 1°, the cuboids no longer sit in this maxima, and only an anti-clockwise rotation decreases the magnetic energy.

It is worth noting that, after jamming, the cuboids are not aligned with the field and their orientation with respect to their center-to-center direction depends only on the geometry of the cuboids and lattice (cf. Figure S2). It can be straightforwardly calculated as *θ_jam_* = sin^−1^ (*l*/*s*_x_), where *l* is cuboid width. In our experiments, *l* = 5 μm, *s_x_* = 13 μm giving *θ_jam_* = 22° and we indeed measured the angle between the cuboids principal axis after rotation and the chain as 22° ± 2° (measured from *n* > 40 cuboids at *θ_A_* = 1°). This angle determines a new transition in the interaction between the colloids when varying *θ_A_*. If *θ_A_* is larger than *θ_jam_*, the rotation does not induce contact, whereas if the angle is smaller or equal to *θ_jam_*, jamming will occur prior to full alignment with the field. This is apparent in Figure 2B((ii),(iii)) at *θ_A_* = 1° and 22°, respectively, where jamming creates readily ordered linear chains whereas at *θ_A_* = 45° and 90°, (Figure 2B(iv),(v)), the rotation of the cuboids does not produce their mutual contact.

**Figure 3:**
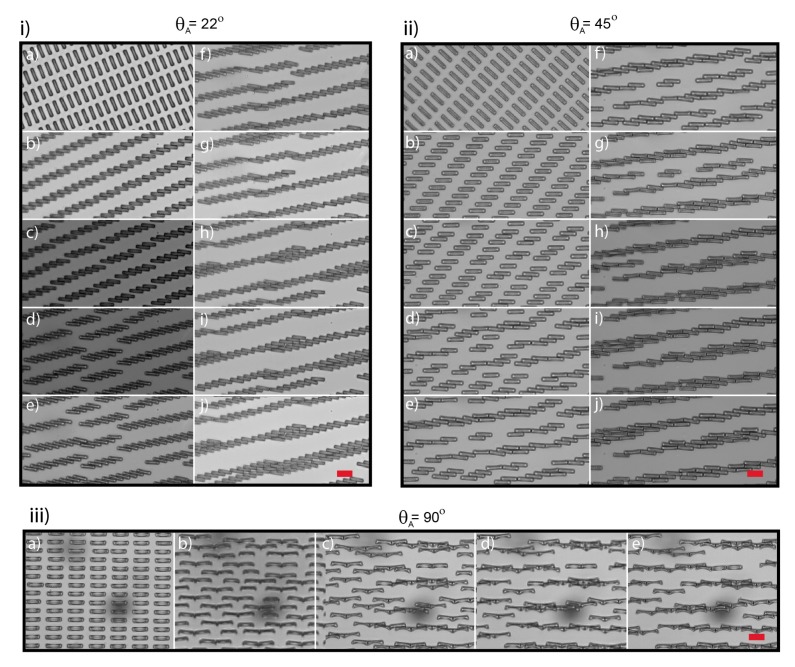
Long term assemblies at *θ_A_* = 22, 45 and 90° (**i**–**iii**), respectively. The time interval between panels (a–j) is 15 and 2.5 s (**i**) and (**iii**), respectively. For (**ii**), the time interval between panels (a–e) is 2 s, 2.75 s between e and f and 10 s between (f–j). Scale bars are 20 μm.

Evolution at longer time scales is markedly different for different angles between the field and the lattice, *θ_A_* (Figure 3). When the field is aligned or close to aligned to the lattice (*θ_A_* = 0° or 1°), the structures do not evolve after formation of jammed linear chains via rotation-induced jamming: Their direction is almost parallel to the magnetic field and repulsive dipole interactions in the perpendicular direction stabilize them against further aggregation. However, when *θ_A_* draws close to *θ_jam_*, the jammed linear structures are formed with an angle oblique with the magnetic field. There is thus a torque acting on the jammed chain to align with the field direction, leading to further assembly, at a larger spatial scale, between the jammed chains themselves (Figure 3i and quantified in Figure S3). Here, the cuboids rotation forms jammed chains that are at angle ~22° with respect to the field in the *x* direction. The chains start to rotate, reducing the angle from 22° ± 1° to 12° ± 3° after 30 s. Full rotation until alignment to the field is however impossible due to the large length scale involved, and smaller chains of 3 to 10 cuboids are formed. The rotation of these chains is further impaired when they touch their neighbors, forming new elongated jammed structures that are more irregular. Part of the frustration in the system is released by the irregularities in the secondary jamming of multiple chains that end in a misalignment of only 10° ± 1° to the field and very regular 2D structures.

For the structures with an angle *θ_A_* well above *θ_jam_*, aggregation at longer time scales occurs in a manner similar to the maturation/aggregation process seen in sparser lattices at *θ_A_* = 0° because the cuboids are able to initially align with the field. Consequently, attractive dipolar forces will aggregate the cuboids. For instance, at *θ_A_* = 45° (Figure 3ii), after rotation, the cuboids aggregate typically in doublets (or triplets) with one of their neighbor in the same row. They then aggregate end-to-end with their nearest neighbor doublets or sole cuboids in the next row. For this particular lattice arrangement, this end-to-end interaction occurs mainly between cuboids of two neighboring rows, shifted by one column. Aggregation continues until elongated structures of remarkably uniform paving are formed. The angle of the elongated structures with the field is small (8° ± 1°) and depends non-trivially on the geometry of the lattice and the minute of the aggregation mechanism. When the cuboids’ principal direction in the lattice is aligned with the field (*θ_A_* = 90°) the cuboids aggregate directly end-to-end into doublets. During this process, the cuboids flip from their bases to reveal the curvature of their side profile. This is likely due to the proximity of the cuboids in the perpendicular direction to the field which are close enough to exert repulsive interactions. As the height of these cuboids is smaller than their width, it is energetically favorable to distance their surfaces from each other, which can be done by flipping. As in the case of Figure 2A(ii), the doublets maturate into long chains. Interaction between segments in adjacent lines that are shifted in the *x* direction can be attractive, and chains are thus formed with cuboids from several lines. Once long chains are formed, however, they remained separated and aligned with the field direction.

## 3. Discussion

### 3.1. Phase Diagram

**Figure 4:**
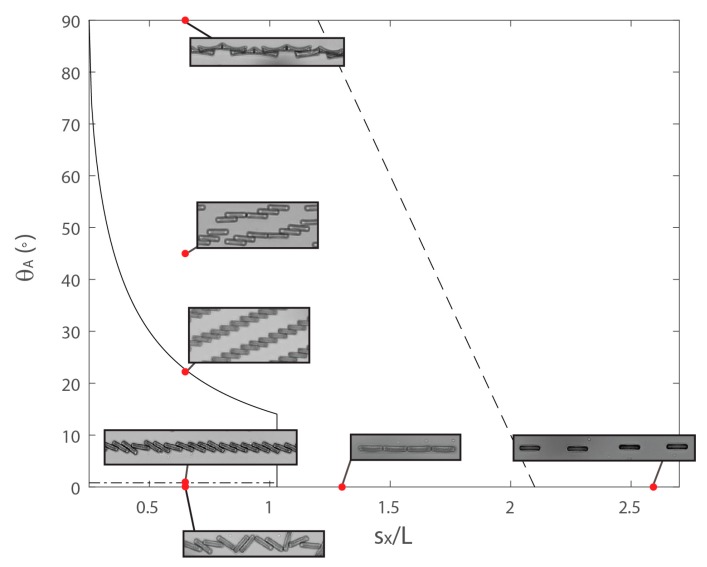
A ‘phase diagram’ diagram displaying the various structures formed as a function of *θ_A_* and the reduced separation, *s_x_*/*L*. The irregular horizontal dashed line is the border between irregular and regular chains (formed from rotation-induced jamming), the black line is the border between rotation-induced jamming and dipole-dipole assembly and the regular dashed line is an estimation of the border between dipole-dipole assembly and non-interaction of the cuboids. The structures shown are those formed at early times.

To summarize our findings, we represent the different mechanisms of aggregation in a ‘phase diagram’ (Figure 4) dependent on the geometric factors: dimension of the cuboids, dimension of the array, and angle between the array and the field. We render the diagram dimensionless by dividing all lengths by *L*. We chose here to limit our study to a constant aspect ratio of the cuboids’ shape (*l*/*L* = 4) and to vary the dimension of the array *s_x_* (keeping the aspect ratio, *s_x_*/*s_y_*, constant). Thus, the two variables on the phase diagram are the angle *θ_A_* and the ratio *s_x_*/*L* that identify two broad assembly processes, rotation-induced jamming, and dipole-dipole assembly. For instance, at low *θ_A_* and low *s_x_*/*L* (bottom left of the phase diagram), the cuboids touch each other before completing alignment with the field, and the assembly in this domain is dominated by jamming mechanisms. When the distance between cuboids is increased (horizontal shift to the right on the phase diagram), there is a transition to a dipole-dipole assembly—the cuboids have enough space to rotate fully until alignment with the field before touching. A straightforward geometrical calculation gives a value at which this transition occurs in our conditions of *s_x_*/*L* = 1.03 (see Figure S2). At low *s_x_*/*L* (i.e., <1.03) and increasing *θ_A_* (vertical shift to the top), the rotation angle needed to fully align with the field decreases so that jamming is avoided at progressively smaller values of *s_x_*/*L* (as calculated from *θ_jam_* = sin^−1^ (*l*/*s_x_*)).

A particular situation is found when the orientation is strictly perpendicular to the field leading to irregular jamming. The transition occurs between *θ_A_* = 0° and 1° and *s_x_*/*L* < 1.03.

Finally, at large separation, the cuboids have enough space to align fully with the field but the dipolar interaction may be not large enough to drive aggregation.

The transitions between rotation induced jamming and dipole-dipole assembly (shown in solid line on the phase diagram), do not depend on the field strength but only on the geometrical parameters of the cuboids and the lattice. In contrast, the transition between dipole-dipole assembly and no interaction of the cuboids (regular dashed line) depends on the field strength.

It is clear then, by adjusting *θ_A_* and lattice parameters, we can select for distinct assemblies. It is significant to note that APPS avoids a bottleneck in self-assembly in that one set of starting components may proceed to form only a limited range of structures. With APPS on the other hand, identical building blocks may be coerced into forming a broad range of architectures by altering their initial arrangement or angle of applied magnetic field, as demonstrated in this communication. Although this phase diagram is not yet fully explored, it already provides strong design principles for assembly. Ultimately, one should be able to assemble a vast host of different particle shapes to build complex and functional architectures.

### 3.2. Towards Micro-Machines

APPS is a new versatile framework with the potential to build customized flexible devices with the elaborate and field responsive parts suitable for high-end applications—particularly in microrobotics. Though lithography approaches can be used directly to make custom designed microstructures, there is a real advantage in fabricating such architectures via the assembly of distinct particles. The former approach yields a single block of rigid material, whereas the latter one can produce a jointed, articulated structure with many more degrees of freedom.

**Figure 5:**
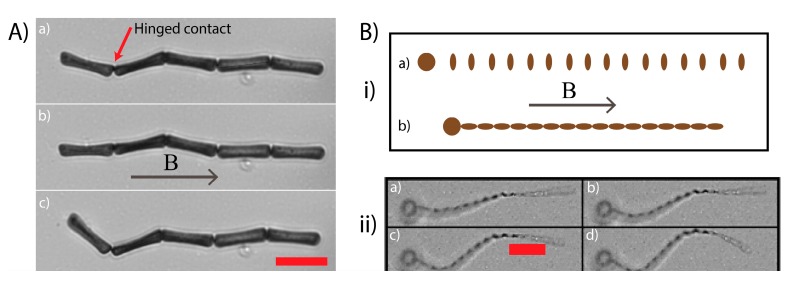
(**A**) A linear chain of cuboids that is flexible due to soft contacts between the cuboids. The initially relaxed chain (a) straightens out to align with an applied homogeneous magnetic field (b) before relaxing again on its removal (c). (**B**) (i) A schematic of the assembly process. The starting arrangement of SP particles: cylindrical tail units and a single spherical head (a). When a magnetic field aligned with the long axis of the arrangement is applied, the tail units align and assemble end to end before connecting with the head (b). (ii) A SP ‘microswimmer’ assembled as depicted in (i) and then flexed with a magnetic field gradient. On application of the field the tail flexes and the panels proceed from (a–d). Scale bars are 20 μm.

The capacity for APPS to build flexible microstructures is demonstrated in Figure 5A showing an ordered chain consisting of five cuboids. The chain, which is initially relaxed, straightens out in the direction of an applied 5 mT field and relaxes again on its removal. Whilst flexibility is noticed at each of the chains contact points, it is apparent that its degree varies considerably across the structure. In particular, the top node (as viewed in the figure) permits far more scope for movement than the other three. This appears to be the result of the particular contact made here; whereas the other contacts are made along the face of the cuboids width, this top contact is at the corners of the width, permitting more lateral movement. Indeed, the other contact points are only flexible because of the cuboids’ concave widths—if the widths were perfectly flat, no motion would be possible. This shows that curvature and/or contact width at a contact point can be manipulated to tune the flexibility of structures formed via APPS. A further way to modulate flexibility of structures formed from APPS would be to integrate polymer links between particles to bind them, as has been done previously in other SP microstructures [37].

We now combine the two stand-out features of APPS, i.e., guiding assembly via bespoke geometric effects and the resultant flexible microstructures formed, to make a jointed microstructure that hold the design features for swimming at low Reynolds numbers (Figure 5B). The starting pattern is intuitive, a large spherical head unit (radius = 10 μm) followed by 16 ovals (long axis = 5 μm, short axis = 2 μm) with a center-to-center separation of 2 μm. On application of a magnetic field, the ovals immediately rotate to align their long axis with it, forming, in a single step, the tail unit. This tail unit subsequently attaches to the head to complete assembly. By applying a field gradient nearby the swimmer, we demonstrate its considerable flexibility. The study of its swimming abilities is however beyond the scope of this article. Instead, we focus on the proof-of-concept of swimmer assembly using APPS—a procedure that can be expanded to facilely fabricate microswimmers to high yields and to specification.

## 4. Materials & Methods

To make the precursor dispersion we mix ETPTA with cleaned and vacuum dried 300 nm diameter silica-coated superparamagnetic (Adembeads) to 33 wt % solid content. At this concentration, the dispersion still flows and can be facilely loaded into micron-sized PDMS wells that template the cuboids and custom parts of the ‘microswimmer’. After loading, the monomer is reticulated with UV light to form the necessary particles. Particles were extracted onto glass microscope slides, embedded in a layer of the polymer, poly(1-vinyl-pyrrolidone-*co*-vinylacetate) (P(VP-VA)), employing the materials and procedure outlined in detail in reference 29 (and as depicted here in Figure 1). A 1:1 mixture of ethanol: ethanediol was employed to dissolve the polymer and act as a viscous carrier fluid for the cuboids. The high combined viscosity of the solvent and polymer allowed retention of the original array pattern for times long enough to permit sample loading on an Axio A1 inverted microscope (Zeiss, Oberkochen, Germany) where the dissolving of the polymer took place simultaneously with application of a magnetic field and imaging. Field application took place using mounted coaxial coils (SBEA, Vitry, Fr coils, Paris, France), with mu-metal cores (length: 40 mm, diameter: 26–88 mm, 750 spires) and imaging with a Neo CMOS high speed camera (Andor Technology, Belfast, UK). The coils were powered by a bipolar operational power supply amplifier 6 A/36 V (Kepco, Flushing, NY, USA) and controlled by LabVIEW software (National Instruments, Austin, TX, USA). A ×40 (Zeiss, NA = 0.65, Oberkochen, Germany) objective was used for microscopic imaging.

## 5. Conclusions

In conclusion, we have shown how the shape and pre-arrangement of micron-sized superparamagnetic particles can be used as reliable handles to guide their aggregation under a homogeneous magnetic field. This work is, to our knowledge, a new concept in self-assembly, one that we label ‘assembly modulated by particle position and shape’ (APPS). We demonstrate this concept on superparamagnetic cuboids, of length, *L* and width, *l*, arranged in rectangular lattices which we direct into distinct assemblies by varying their center-to-center separation along the *x* coordinate, *s_x_*, and angle of the lattice, *θ_A_,* relative to the magnetic field.

We encapsulate the influence on of these variables in a ‘phase diagram’ that differentiates two aggregation pathways: rotation-induced jamming and dipole-dipole assembly. The former takes place at smaller separations of the cuboids such that their immediate rotation under field leads to 1D, jammed linear chains, whereas the latter occurs at greater separations and is defined by dipole-dipole assembly after the rotation of the cuboids, producing 2D space filling structures or linear chains of cuboids assembled end to end. Significantly, the boundary between these pathways is insensitive to the strength of the applied field. We also identify a non-interaction region of our ‘phase diagram’ at larger center-to-center separations where the cuboids rotate but do not assemble. The boundary between this region and the region characterized by dipole-dipole assembly will be a function of field strength along with the other variables identified in the proceeding.

We also demonstrate that structures formed through APPS have the potential to be flexible and suggest that judicious choice of shape and arrangement can lead to microstructures with complex actuation modes. We strongly support this suggestion by designing a starting arrangement of shapes that assemble easily into a microstructure with essential design features for swimming at low Reynolds numbers.

We emphasize that the results presented here are to communicate the grand potential of the APPS process and are but the starting point for significant further work to fulfill said potential. In this study, we focused on simple patterns of cuboids, but complex and non-regular arrangements will be explored in the future—presenting a zoo of different shapes—with an ultimate aim to decipher rules that link the starting arrangement and shapes of the particles to the structures they form under magnetic fields.

## Supplementary Materials

The following are available online at www.mdpi.com/1996-1944/10/11/1291/s1. Figure S1: The average chain length as a function of time under homogeneous magnetic fields at 5 mT. Starting conditions of *θ_A_* = 0° and *s_x_* = 26 μm, Figure S2: Details on phase diagram calculations, Figure S3: Evolution of the principal angle of the cuboids with the applied magnetic field from starting conditions of *θ_A_* = 22° and *s_x_* =13 μm.
