# Supplementary material for: Assembly Modulated by Particle Position and Shape: A New Concept in Self-Assembly
Source: Materials (Basel). 2017 Nov 10;10(11):1291. doi: 10.3390/ma10111291 (PMC5706238; doi:10.3390/ma10111291)
Supplement: Supplementary file 1 [file materials-10-01291-s001.pdf]

Supplementary Material

S1: Chain maturation

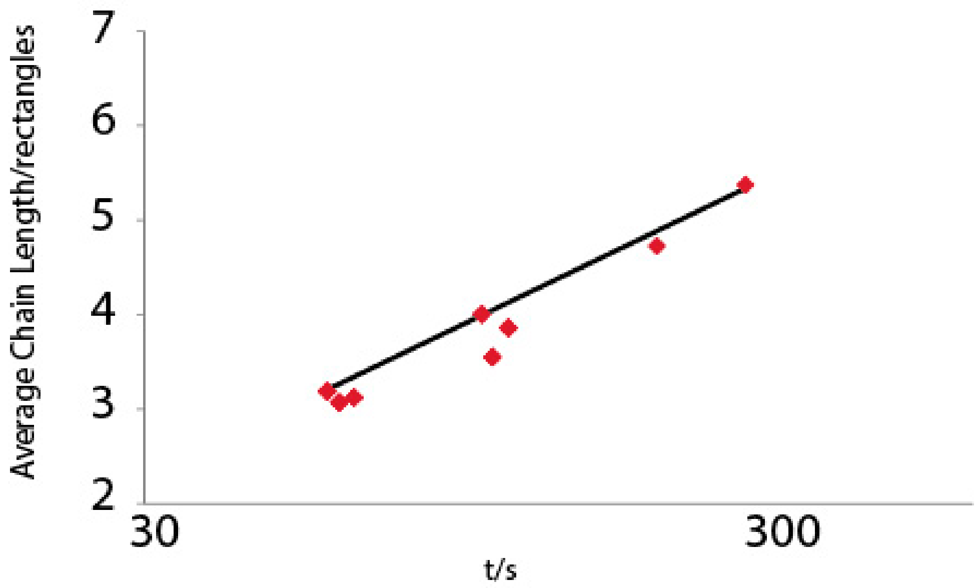

Figure S1. *The average chain length as a function of time under homogeneous magnetic fields at 5 mT. Starting conditions of θ_A=0° and s_x = 26 µm.*

S2: Details on phase diagram calculations

The diagram of Figure 4 shows the frontiers between different modes of aggregations that we evidenced. The limits are theoretical limits calculated from the geometry of the lattice and the cuboids. All distances have been rendered a-dimensional by dividing them by the length of the cuboid L. The separation between cuboids in the transverse direction s_x is a parameter of the diagram. The two other distances, $l=L/4$ the width of the cuboids, and s_y the distance between cuboids in the longitudinal direction have been fixed in relation to the others. Other choices of these relationships would lead to changes in the diagram.

The second parameter is θ_A the angle between the direction transverse to the cuboids and the direction of the magnetic field.

When θ_A = 0°, the cuboids rotate 90° and the distance between the centers of the cuboids determines if they will contact and jam or stay separated. The transition occurs when the distance s_x is longer than the diagonal of a cuboid. The diagonal is the longer length in the cuboid, and at the transition the corners will touch during rotation. For cuboids of length L and width l for which L= 4 l, the diagonal is $\sqrt{L^2 + l^2} = L\sqrt{1 + \left({}^1\!/_4\right)^2} = 1.03\,L$. Hence the transition occurs for s_x/L = 1.03.

When the cuboids are in contact through this rotation, one can calculate geometrically the angle (alpha) between the cuboids main direction and the line going through their center. If the torque acting on the cuboids rotates them so that the angle between their principal axis and the line going through their center is above alpha, the cuboids will not touch. This angle is $\sin\alpha = \frac{l}{s_x}$, as can be seen on the sketch below. For these specific cuboids $\alpha = \sin^{-1}\left({}^{L}\!/_{4s_x}\right)$. Hence the frontier calculated geometrically between the torque mediated assembly and other type of assembly consists of a curve calculated from $\sin^{-1}$ and a vertical line at s_x/L = 1.03.

The two limits join at $s_x/L = 1.03$ and $\theta_A = 14°$.

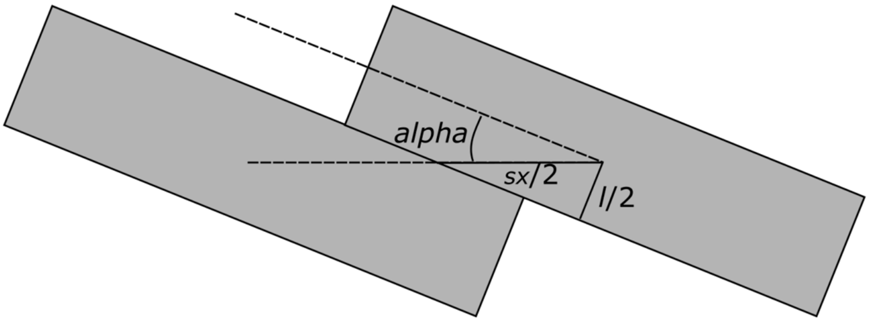

Figure S2. *Details on phase diagram calculations.*

When the cuboids do not contact via rotation, they will contact by dipole-dipole interaction if the distance between their borders are sufficiently small (attractive aggregation). Contrary to the transition between torque-mediated and attractive aggregation, the transition between attractive aggregation and no-aggregation depends strongly on the field strength. The dashed line separating these regions is meant as a general separation rather than a precise calculation. We chose for the limit at $\theta_A = 0°$ an arbitrary point between the observed aggregation and no aggregation and used the distance between cuboids in this case to calculate the limit at $\theta_A = 90°$.

S3: Evolution of the principal angle of the cuboids with the applied magnetic field during assembly

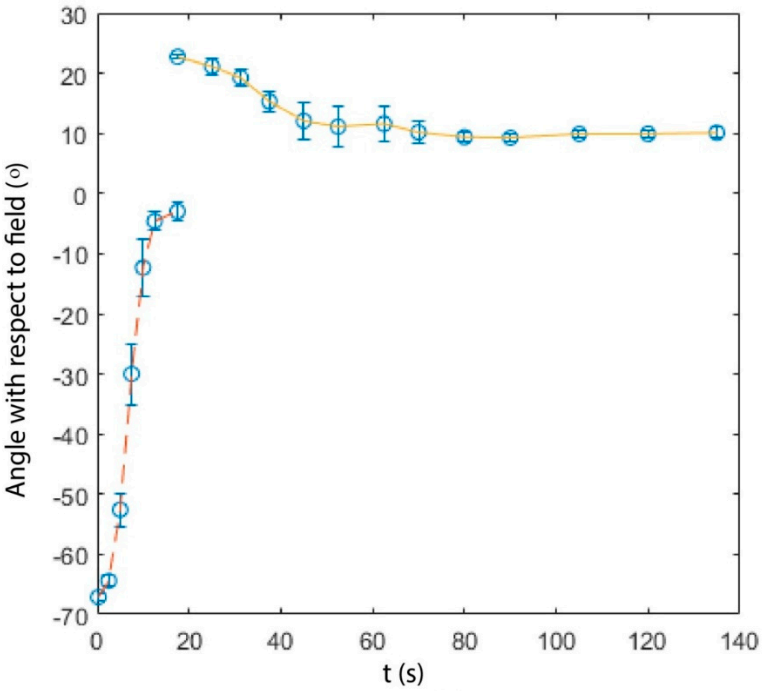

Figure S3. *Evolution of the principal angle of the cuboids with the applied magnetic field from starting conditions of* θ_A*=22° and* s_x*=13 µm.*
